# Association of Muscle Strength and Fasting Glucose Levels among the Elderly in South Korea: Cross-Sectional Pilot Study

**DOI:** 10.18502/ijph.v49i7.3601

**Published:** 2020-07

**Authors:** Kyoungkyu JEON, Dong-il KIM

**Affiliations:** 1. Division of Sport Science, Incheon National University, Incheon, Republic of Korea; 2. Sport Science Institute, Incheon National University, Incheon, Republic of Korea; 3. Division of Health and Kinesiology, Incheon National University, Incheon, Republic of Korea

## Dear Editor-in-Chief

Aging is closely related with increased levels of obesity and reduced levels of lean body mass (1). Decreased physical activity due to muscle loss is very important cause of falls among the elderly people (2). The increase in physical activity can increase muscle strength, physical fitness, and body muscle mass and reduce the risk of diabetes (3). However, the effect of increasing muscle strength through physical activity on diabetes has not been elucidated well among the elderly in South Korea.

Therefore, we aimed to investigate the association of muscle strength levels and fasting glucose levels with the risk for diabetes in the elderly based on the data obtained from this pilot study in South Korea.

We conducted a cross-sectional pilot study that enrolled 43 elderly people (4 males, 39 females) from Eunpyung Municipal Welfare Center in Seoul, Korea who met our inclusion criteria (Table 1).

**Table 1:** Participant characteristics

*** Variables ***	*** Female (n=39) ***	*** Male (n=4) ***	*** P-value ***
Age (yr)	73.76±4.65	73.50±6.85	0.916
Body Composition			
Height, cm	151.15±4.55	168.20±3.88	<0.001
Weight, kg	59.12±8.69	69.40±8.40	0.055
BMI, kg/m ^ 2 ^	25.84±2.77	24.70±2.87	0.497
Blood Analysis			
Fasting Glucose, mg/dl	90.92±8.93	86.00±13.73	0.323
Fitness Test			
30-sec Chair stand, n	13.43±3.01	15.75±1.50	0.140
Push up, n	13.07±10.54	7.5±6.40	0.309

Data are presented as mean ± standard deviation. BMI: Body mass index

This study was approved by the Ministry of Health and Welfare, Republic of Korea, and written informed consent was obtained from all participants. Body composition was measured using In-Body (IHU070R, Biospace, Seoul, Republic of Korea). Physical fitness was evaluated based on the 30-second chair stand test as a measure of lower-body (LB) strength and the push-up test as a measure of upper-body (UB) strength, designed for the elderly. Blood samples from the elderly were collected after a 12-h fasting period and analyzed for fasting glucose levels (ADVIA 1650, Siemens, Tarrytown, NY, USA).

Statistical analyses were conducted analyzed using SPSS, version 21.0 for Windows (SPSS Inc., Chicago, IL, USA). Independent *t*-tests were used to compare between high and low levels of upper and lower body strength. Furthermore, ANCOVA were used to compare fasting glucose levels among the groups while adjusting for the effect of age and obesity. The results were considered significant for *P*<0.05.

Fasting glucose levels were significantly lower in the high levels LB strength group than in the low levels LB strength group (*P*=0.03). The subjects were categorized into the following four groups based on their levels of UB and LB strength: 1) High LB and high UB strength; 2) High LB and low UB strength; 3) Low LB and high UB strength; and 4) Low LB and low UB strength. Differences in the fasting glucose levels among the four groups were determined while adjusting for the effect of age and obesity (Table 2). Fasting glucose levels were significantly lower in the high LB & high UB strength group compared to that in the low LB & low UB strength group (*P*<0.05).

**Table 2:** Fasting glucose levels according to levels of upper-body and lower-body strength

^*^ *** Model 1: *** * Levels of upper-body and lower-body strength on fasting glucose levels *						
	** LB Strength **		***P*-value **	** UB Strength **		***P*-value **
	** High **	** Low **		** High **	** Low **	

No.	19	24		23	20	
Fasting Glucose, mg/dl	87.10±5.59	93.12±10.91	0.035	88.95±6.62	92.20±11.71	0.262

^*^ ** Model 2: ** * Combined association of upper and lower-body strength on fasting glucose levels *				
	** High LB Strength **		** Low LB Strength **	
	** High UB strength **	** Low UB strength **	** High UB strength **	** Low UB strength **

No.	14	5	8	15
Fasting Glucose, mg/dl	86.18±2.53	88.28±4.29	90.55±3.19	94.57±2.37 ^*^

Data are presented as mean ± standard deviation (Model 1) and error (Model 2), LB: Low-body, UB: Upper-body, Model 2: The data represent result from ANCOVA analyses controlling for age and BMI,

* *p* < 0.05 difference between 1st group and 4th group

Besides, we investigated the association of LB and UB muscle strength and fasting glucose levels among the elderly in South Korea. We found that the levels of muscle strength were associated with fasting glucose levels. Moreover, we observed a significant decrease in fasting glucose levels in the high LB and high UB strength group compared to the low LB and low UB strength group(*P*<0.05). These results suggest that increasing muscle strength through physical activity emphasizes the importance of the association of fasting glucose levels in the Korean elderly people. Generally, as participation in regular physical activity increases, muscle strength increases, while the incidence of risk factors associated with impaired glucose metabolism decreases in elderly people (4).

This study had a limitation. It is a cross-sectional pilot study with a small sample size of the elderly persons in South Korea. Additional studies with an adequate sample size calculated based on the effect size detected in this study must be conducted.

In conclusion, muscle strength is significantly associated with the fasting glucose levels. Therefore, this study provides evidence that increasing muscle strength by regular resistance exercise may improve the fasting glucose levels in the elderly people in South Korea.
